# Software for interpreting cardiopulmonary exercise tests

**DOI:** 10.1186/1471-2466-7-15

**Published:** 2007-10-23

**Authors:** Robert M Ross, David B Corry

**Affiliations:** 1 Department of Medicine Baylor College of Medicine, Houston, Texas, 77030, USA

## Abstract

**Background:**

Cardiopulmonary exercise testing (CPET) has become an important modality for the evaluation and management of patients with a diverse array of medical problems. However, interpreting these tests is often difficult and time consuming, requiring significant expertise.

**Methods:**

We created a computer software program (XINT) that assists in CPET interpretation. The program uses an integrative approach as recommended in the Official Statement of the American Thoracic Society/American College of Chest Physicians (ATS/ACCP) on Cardiopulmonary Exercise Testing. In this paper we discuss the principles behind the software. We also provide the detailed logic in an accompanying file (Additional File 1). The actual program and the open source code are also available free over the Internet at . For convenience, the required download files can also be accessed from this article.

**Results:**

To test the clinical usefulness of XINT, we present the computer generated interpretations of the case studies discussed in the ATS/ACCP document in another accompanying file (Additional File 2). We believe the interpretations are consistent with the document's criteria and the interpretations given by the expert panel.

**Conclusion:**

Computers have become an integral part of modern life. Peer-reviewed scientific journals are now able to present not just medical concepts and experimental studies, but actual functioning medical interpretive software. This has enormous potential to improve medical diagnoses and patient care. We believe XINT is such a program that will give clinically useful interpretations when used by the medical community at large.

## Background

In May of 2003 a joint committee of the American Thoracic Society and The American College of Chest Physicians (ATS/ACCP) published a Statement on Cardiopulmonary Exercise Testing (CPET) [1]. One of the stated primary purposes of the document was to facilitate interpretation and clinical application of CPET. However, other authors have pointed out that the data generated from CPET are easily the most difficult set of results a director of a pulmonary function laboratory has to interpret. They also give the opinion that the resources available to help physicians in interpretation are limited. They state that although the ATS/ACCP statement is comprehensive, it must be approached with "zeal" in order not to be overwhelmed [2]. That is, interpreting these tests is often difficult and time consuming; requiring significant expertise. This has the potential to limit its usefulness.

In keeping with the Committee's goals, we have written a computer program called XINT [3] to assist clinicians in the interpretation of CPETs. The program provides uniform interpretations of cardiopulmonary exercise physiology data that cover a broad range of physiologic abnormalities. The interpretations conform to the recommendations of the ATS/ACCP and are readily understood by the specialist and non-specialist alike. XINT does not use a simple logic-tree but an integrative approach to CPET interpretation as recommended in the ATS/ACCP document.

In the first section of the report created by XINT, the findings are presented in a tabular format and compared to normative data. The program then discuses each pertinent CPET variable. It then integrates them where appropriate into "key findings" somewhat similar to the "usual cardiopulmonary exercise response patterns" described in table 18 and figure 10 in the ATS/ACCP document. Finally, XINT describes the degree of impairment that might be expected.

In this paper we will briefly discuss some of the medical and physiologic concepts behind XINT. We provide few references, as they are well detailed in the ATS/ACCP document. The detailed logic underlying its interpretative strategies is too extensive to present in this article but is available as an on-line supplement (Additional File 1). Further, the actual source code is open source and can be read directly, using a simple text editor. To demonstrate its utility and to validate XINT, the software has been used to interpret the same data sets presented in the ATS/ACCP document. The results are presented in Additional File 2. We believe the computer-generated reports are consistent with the interpretations given by the expert panel in that document.

## Implementation

XINT was written in the programming language ActiveState Tcl, which is provided free and is open source (a). This platform provides ease of dissemination and use of the program. XINT runs in both a Windows environment and on a Macintosh. The source code may be changed with a simple text editor and does not require compiling. XINT and the appropriate version of Tcl are both free and available on the Web at the XINT website [3]. They are also available from this document.

### Required Fields

As well as demographic information, XINT requires the following variables to create an interpretative report: Age, height, weight and gender; Forced Expiratory Volume in 1 Second (FEV1) and forced Vital Capacity (FVC) or Vital Capacity (VC) if larger; Maximum oxygen uptake (VO2 max) in ml/kg/min or ml/min; Anaerobic Threshold (AT) in ml/kg/min or ml/min (if there was one); Maximum heart rate achieved; Ventilatory equivalent of oxygen (VE/VO2) at mid-exercise; Maximum tidal volume achieved and Maximum ventilation achieved. The Heart rate equivalent of oxygen (HR/VO2) is also required but will be estimated if it is not entered. XINT will also evaluate many other variables if entered. Table 1 lists all optional and required variables for XINT and pertinent information related to each. The variable names listed in the table are those used in the software code.

**Table 1:** XINT Variables

**Variable**	**Description**	**Variable**	**Description**
**Demographic Information**			

firstName	Patient's first name	lastName	Patient's last name
patId	Patient's identification number	tDate	Date of test
age *	Age in years	sex *	Male or female
height *	Height in inches	weight *	Weight in pounds
baroPr	Barometric pressure (mmHg)	exTimeMin	Exercise time (minutes)
exTimeSec	Exercise time (seconds)	termReason	Reason for terminating the CPET
TermBy (Tt)	Test terminated by the observer	doc	Doctor's name
lowHrResp	Heart rate response low? (y/n)	pcntBf	Body mass index (BMI)

**Spirometry Variables**			

preFev1 *	FEV1 before exercise	preFvc *	FVC before exercise
preFev1Fvc	FEV1/FVC before exercise	preMvv	Maximum voluntary ventilation before exercise (MVV)

**Physiological Variables**			

maxVo2Kg *	Maximum (or peak) oxygen uptake (ml/kg/min)	maxVo2M1 *	Maximum (or peak) oxygen uptake (ml/min)
maxPMph	Maximum treadmill speed (mph)	maxPE1ev	Elevation of treadmill (%)
maxR *	Respiratory exchange ratio (R) at maximum exercise	atKg	Anaerobic threshold (AT) (ml/kg/min)
atM1	Anaerobic threshold (AT) (ml/min)	maxP	Maximum power output (watts)
wattsVo2	Slope of VO2 (ml)/power(watts)	maxHr *	Maximum heart rate obtained during CPET
hrVo2	Slope of heart rate/VO2(L) mid-exercise	restHr	Resting heart rate
restSBp	Resting systolic blood pressure	restDBp	Resting diastolic blood pressure
exSBp	Maximum exercise systolic blood pressure	exDBp	Maximum exercise diastolic blood pressure
midVeVo2 *	VE/VO2 at mid exercise	maxVeVo2	VE/VO2 at maximum exercise
midVeVCo2	VE/VCO2 at mid exercise	maxVeVCo2	VE/VCO2 at maximum exercise
maxTv *	Tidal volume at maximum exercise	restVe	Ventilation (VE) at rest
maxVe *	Ventilation at maximum exercise	breatheRsrv	Breathing reserve
rraPac02	Arterial PCO2 before exercise	rraPa02	Arterial PO2 before exercise
rraHco3	Bicarbonate (HCO3) before exercise	rraO2Sat	02 saturation before exercise
exPaCo2	Arterial PCO2 at maximum exercise	exPa02	Arterial PO2 at maximum exercise
exHco3	Bicarbonate (HCO3) at maximum exercise	ex02Sat	O2 saturation at maximum exercise
maxAa	(A-a) O2 difference at maximum exercise	maxVdVt	VD/VT at maximum exercise

Note: In the source code, variables prefixed with a "p" indicate predicted values and variables prefixed with "pp" indicate percent of predicted values. * indicates required variables. Others are optional and will be interpreted if entered.

### Reference Values

Table 2 lists the reference equations for selected variables used by the interpretation program [4-7]. Constant predicted values are presented on the XINT data input screen or in the discussion of each variable.

**Table 2:** Reference Equations Used for interpretation by XINT.

Spirometry [4]:
FEV1 Male: (0.132 × height (cm)) - (0.027 × age (yrs)) - 4.20
Female: (0.069 × height (cm)) - (0.021 × age (yrs)) - 0.794
FVC Male: (0.165 × height (cm)) - 0.029 × age (yrs)) - 5.45
Female: (0.094 × height (cm) - 0.022 × age) - 1.774
Estimating Vo2 From External Power Output:
Treadmill (walking) [5]:
VO2max (ml/kg/min) = maximum speed (Mph)* (262.5 + 17.5* elevation)/60
Cycle ergometer:
VO2 max (ml/min) = maximum Watts *11 + 300
Estimated Heart Rate-Vo2 Slope (Hr/Vo2) If Not Entered [6]:
Male: (maxHR - 63)/maxVo2M1/1000
Female: (maxHR - 73)/maxvo2M1/1000
Maximum Ventilation:
MVV if entered, else FEV1 × 35
Maximum Heart Rate:
202 - (0.72 × Age (yr))
Maximum Predicted Oxygen Uptake (Ml/Kg/Min) [7]:
Male: 43.2 - (0.255 × age)
Female: 42.3 - (0.33 × age)

Reference values are a challenging area for CPET. Table 12 in the ATS/ACCP document lists various studies of normative or reference values and the studies' limitations. The committee concludes that none of the studies fulfill the requirements for "optimal" reference values. XINT attempts to minimize these problems by using an integrative approach and cut-off values that are clinically meaningful. For example, an FEV1/FVC ratio of 68% is used for "airflow obstruction" because although there may be airflow obstruction above this value it usually does not lead to significant exercise limitation. Mild exercise limitation is not commented on by XINT as it may be within normal limits for the general population. Further, if an individual has normal exercise capacity many minor physiologic results which could represent minimal abnormalities under different conditions are ignored. The integrative approach depends on many factors. The logic XINT uses is similar to that of a human expert in CPET interpretation. The accompanying file, Additional File 1, provides a detailed discussion of the XINT logic.

Another way the program addresses the complexity of reference values is that, if desired, the user can change any of the predicted values directly on the input screen.

### CPET Variables Assessed

#### Maximum Oxygen Uptake (VO2max or VO2peak)

For the purposes of interpretative program we do not differentiate between VO2max and peak oxygen uptake (VO2peak). VO2max is used as the determinant of overall cardiopulmonary/metabolic function. The XINT interpretation divides VO2max into functional categories not just based upon comparison to average values but also on the physiological requirements for normal activities of daily living. XINT also adjusts for significant obesity. If Body Mass Index (BMI) is greater than 40 and the VO2 max is reduced the program determines what VO2 max would be based on a more normal BMI.

#### Maximum External Power Output

There is a relatively linear relationship between external power output, such as treadmill walking (speed and elevation) or cycle ergometry (Watts) and oxygen uptake. If the relationship is abnormal, XINT assumes there is a quality control issue, unless VO2max is significantly reduced. Then, an abnormality of oxygen delivery kinetics may be suggested.

#### Rate of Perceived Exertion (RPE)

Rate of Perceived Exertion (RPE), also known as the Category Ratio (CR)-10 scale is a psycho-physiological scale that allows patients to document how difficult they perceive a physiological stress such as a CPET to be. When malingering or secondary gain issues can be excluded, RPE has been found to be a useful indicator of physiological stress and exercise limitation.

#### Heart Rate during Exercise

Since cardiac output increases in a relatively linear fashion with external power output and stroke volume becomes constant relatively early in exercise, the slope of the heart rate response to exercise represents an index of stroke volume. This slope should be measured in the mid portion of exercise, after the stroke volume becomes stable. This is termed the heart rate equivalent of oxygen (HR/VO2). Since under normal conditions the cardiac output increases about 5L/min for every increase of 1 L of VO2, a normal stroke volume of about 100 ml would lead to a HR/VO2 of about 50 beats/L VO2. It would be somewhat higher for smaller people as they have smaller stroke volumes.

With normal exercise limitation, as well as for people who are unfit, the heart rate tends to reach an individually determined maximum, which is related to age. With significant cardiac disease, the heart rate may or may not reach maximum predicted depending upon the physiological factors that lead to exercise limitation. Cardiac conduction abnormalities or various drugs such as beta-blocking agents may affect the HR/VO2 relationship and the maximum heart rate attained during a CPET.

#### Anaerobic or Ventilatory Threshold (AT)

The AT indicates the onset of cardiovascular and/or metabolic "strain" and predicts exercise limitation due to these factors at about twice the VO2 where the AT occurs. A low VO2 max with an AT occurring at about 50% of the actual VO2max usually indicates a cardiocirculatory/metabolic exercise limitation. If there is another cause for significant exercise limitation, the AT might still occur but at a greater percentage of the actual VO2 max.

#### Blood Pressure and Electrocardiogram (ECG)

A CPET is also a cardiac stress test. XINT comments on blood pressure and ECG changes.

#### Spirometry

Spirometry is analyzed for significant pathology that might be expected to lead to exercise limitation. Therefore, minimal obstruction or restriction is not commented on.

#### Ventilation during Exercise

Under aerobic conditions below the AT, there is approximately 25 liters of ventilation required for every liter increase of VO2. That is the ventilatory equivalent of oxygen (VE/VO2) at the nadir in the VE/VO2 vs. VO2 curve is approximately 25. High VE/VO2's indicate increased dead space ventilation and/or alveolar hyperventilation. A low VE/VO2 usually indicates alveolar hypoventilation.

Tidal volume usually reaches about 60–65% of the individual's actual FVC relatively early in exercise. It then stays constant thereafter.

Maximum ventilatory ability is approximately 35 times the individual's actual FEV1. Severe respiratory pathology may cause a ventilatory limitation to exercise. If the maximum ventilation achieved is 95% Predicted or greater, there is considered to be a ventilatory limitation. Ventilatory Reserve is considered to be significantly diminished if the maximum ventilation achieved is above 85% Predicted.

#### Blood Gas Related Calculations

CPETs may be performed with or without blood gases or other assessments of oxygen saturation (O2sat) and arterial carbon dioxide level (PaCO2). XINT is equipped to handle these various combinations. The confidence of the statements produced is based on the quantity and quality of the data.

### Acid-Base

#### Acid-Base calculations are made based upon the equation

Hydrogen ion concentration (nanomoles) = 24.5*PaCO2 (mmHg)/bicarbonate (mg %).

A normal pH of 7.40 is about 40 nanomoles. XINT uses a drop in bicarbonate of 3.5 mg% or greater as evidence of lactic acidosis and greater than 2 mg% as mild lactic acidosis.

#### Alveolar Ventilation and Gas Exchange

An elevated PaCO2 during exercise generally represents pulmonary pathology with a markedly reduced ventilatory capacity.

CPET is extremely useful in evaluating gas exchange, or the matching of pulmonary ventilation and pulmonary perfusion (V/Q). Virtually any pulmonary parenchymal disorder (alveolar, interstitial, small airway or vascular) will lead to a less than optimal distribution of V/Q ratios during exercise. This can be detected during CPET by finding increased "dead space" (VD/VT) or increased "shunt" manifested by a widened Alveolar-arterial oxygen ((A-a) O2) difference and arterial desaturation. Unfortunately, the magnitude and proportions of dead space and shunt lack the sensitivity and specificity required to diagnose specific diseases [8]. However, pulmonary vascular disease and, to a lesser extent, congestive heart failure selectively lead to a high dead space, while interstitial pulmonary fibrosis generally results in a significant shunt with variable increases in dead space.

Dead space is high in everyone during tidal breathing, so cannot be reliably evaluated during at low levels of exercise where ventilation is low.

If VD/VT at maximum exercise is greater than 0.3 and ventilation (VE) is 20 L/min or more, it is assumed there are increased areas of high V/Q or dead space. If the exercise (A-a) O2 difference drops by 10 mmHg or more compared to rest and the exercise partial pressure of oxygen (PaO2) is 60 mmHg or less, or if the arterial O2sat during exercise drops 4% or more from resting values and is less than 90%, then it is assumed there are increased areas of low V/Q or shunt.

### Data Discussion

After analyzing each of the CPET variables, XINT attempts to arrive at clinically useful interpretations of exercise capacity, pulmonary and cardiovascular pathology, and degree of impairment.

The program first discusses maximum oxygen uptake compared to average normative values for age and gender. Next, it reviews why the test was terminated. It then reports any pertinent ECG findings. Subsequently, key factors associated with Obstructive Pulmonary Disorders or Non-Obstructive Cardiopulmonary Disorders are commented on. Lastly, XINT estimates impairment if exercise capacity is diminished.

### Key Factors Associated With Significant Airflow Obstruction

Exercise may induce asthma. Patients with airflow obstruction may terminate exercise due to lack of ventilatory reserve. However, they may also terminate exercise in association with airflow obstruction due to many other factors. These include increased work-of breathing due to mechanical changes in the lung, alveolar hypoventilation and gas exchange abnormalities, which increase the ventilatory requirements for any required VO2. Any of these abnormalities can contribute to dyspnea and reduced exercise capacity. These factors can also potentially increase the cardiac output requirements for any level of VO2 [9].

Airflow obstruction is assumed if the FEV1/FVC ratio is 68% or less. The factors the program then looks at are as follows:

- If the FEV1/FVC ratio is 68% or less and the FEV1 is 50% or less predicted there is physiologically significant airflow obstruction.

- If the exercise (A-a) O2 difference on room air is 30 mmHg or greater or the exercise arterial oxygen saturation is less than 90% and drops 4 or more from rest there is exercise hypoxemia.

- If the PaCO2 is greater than 45 mmHg there is alveolar hypoventilation.

### Key Factors Associated With Cardiopulmonary Disorders Without Airflow Obstruction

An FEV1/FVC ratio above 68% is chosen as the cut-off for non-obstruction because this represents either no airflow obstruction or, at most, minimal-to-mild airflow obstruction at which level there should not be a major reduction in exercise capacity solely due to airflow obstruction.

Due to the complexity of factors leading to mild-to-moderately reduced exercise tolerance, if VO2max is 70% Predicted or greater, no specific concluding comments are made for non-obstructive disorders. However, the abnormal physiological findings are reviewed in the discussion of the CPET variables.

Cardiopulmonary disorders unassociated with airflow obstruction can lead to reduced exercise capacity through mechanical factors and gas exchange abnormalities leading to increased work-of-breathing, increased work-of-the-heart and/or metabolic abnormalities.

The physiological abnormalities evaluated are:

- If the exercise arterial oxygen saturation is less than 90% and drops 4 or more from rest there is hypoxemia or "shunt".

- If VD/VT at maximum exercise is 0.3 or greater and ventilation at maximum exercise is greater than 20 L/min there is increased "dead space".

- A low stroke volume is assessed based on a review of maximum heart rate achieved, HR/VO2, drop in bicarbonate level and whether there is an AT.

An isolated low stroke volume with low exercise capacity and no gas exchange abnormalities or significant lung disease suggests left ventricular disorders including unfitness. If there is a significant increase in dead space only associated with a reduced stroke volume and low exercise capacity, congestive heart failure or pulmonary vascular abnormalities are suggested. If there is increased shunt, with or without increased dead space, interstitial/pulmonary parenchymal disorders are suggested which may, or may not, be associated with heart disease depending on the assessment of stroke volume.

### Miscellaneous Statements

- If the maximum ventilation achieved is 95% of predicted or greater and VO2max is not above normal then there is a ventilatory limitation to exercise.

- If there is a significantly low VO2max and a low oxygen cost relative to the external power output then there may be abnormalities of oxygen transport.

- If there is a high ventilatory response to exercise with no gas exchange abnormalities and no cardio-circulatory limitation, this suggests the possibility of anxiety.

- If VO2max is reduced but there is no clear-cut limiting factor and no significant physiological abnormalities then there is no obvious cardiovascular or pulmonary limitation.

- If VO2max is 85% predicted or greater and there is a normal cardio-circulatory limitation to exercise then it is a functionally normal CPET.

### Impairment

An estimate of the patient's impairment is given if VO2 max is less than 80% predicted and there is evidence of a cardiovascular or ventilatory limitation. The patient's level of impairment is presented in METs (1 MET = 3.5 ml/kg/min). This unit is commonly used in work physiology. The working MET level for the patient is the lower of the patient's anaerobic threshold or 50% of VO2 max if there is a ventilatory limitation to exercise. Various studies have shown that this level of exercise can be sustained for prolonged periods [10].

### Reports and Data Handling

The report generated by XINT when the "report" button is pressed on the demographic screen, generally comes up in a text editor. The report can then be edited and saved by giving it a file name chosen by the operator. Also, every time the "report" button is pressed the program creates a file called "cpxrpt.txt" in the cpx folder. If the report does not come up in a text editor, before creating a new report, the operator should go to the cpx folder and rename cpxrpt.txt as desired. This file can be edited, saved or sent to the patient's electronic record if desired.

All of the saved patient data is kept in the cpx folder in a file called "cpxtests.txt". From the "load" button on the program any previously saved test may be retrieved. The program allows for saving partial data as well. However, the report function will not work without all the required data entered. Users should remember to press the "save" button after they enter data, even if they have created a report. Otherwise the data will have to be re-entered.

XINT also allows export and import of a patient's data. To create an export file first enter or load the patient's data onto the XINT entry screen. Next, from the entry screen choose "export" from the pull-down list under "file" at the top left. A file called "cpxexport.txt" is created in the cpx folder. This file may then be renamed or sent where desired as appropriate. To import patient data a file called "cpximport.txt" must be placed in the cpx folder. It has the identical structure as cpxexport.txt, just a different file name. From the entry screen choose "import" from the pull-down list under "file" at the top left. The data will then appear in the on-screen fields. From there the data may be edited and saved. A report can also be generated.

## Results

To test the utility of XINT for CPET interpretation, in a non-biased way, we chose the five case study examples provided in the ATS/ACCP Statement on Cardiopulmonary Exercise Testing [1]. The computer generated interpretations were derived strictly from the data taken from these examples. The reports are presented in Additional File 2. The actual diagnoses are shown in parentheses prior to each interpretation.

We believe the software-derived interpretations are substantially similar to those given by the expert group thus validating this program for a diverse group of non-selected medical disorders.

## Discussion

CPET is increasingly being used due to advances in technology and an increased awareness of its importance in the clinical assessment of patients with diverse medical problems. Despite the rising importance and utility of CPET, the difficulty inherent in interpreting the large data sets generated by even limited studies represents a significant deterrent for both ordering and interpreting CPET. As it has in many areas of science and other complex endeavors, software will undoubtedly become useful for facilitating medical diagnoses and implementing appropriate therapies. XINT represents a substantial beginning in medical interpretive software to assist in patient care. It utilizes an integrative approach similar to the way a human expert does, without reliance on simple algorithms or a logic tree. It reviews the data, describes meaningful abnormalities, and then attempts to summarize key findings.

It is well recognized that even among experts, there is rarely uniform agreement when evaluating complex data such as that presented in the case studies provided in the results section. Yet, the XINT-based interpretations were comparable to those of the expert panel and in each example the program included the actual diagnosis, albeit often within a range of diagnoses. We currently use XINT at several Baylor College of Medicine-affiliated hospitals to provide CPET interpretations for actual patients and find them to be consistently useful and meaningful.

## Limitations

Although XINT is a helpful tool for creating a uniform, clinically significant interpretation of CPET data, it is not a panacea. Many factors can affect its usefulness and accuracy. The program output is highly dependent on the quality of the data collection and the human determination of various critical CPET variables. XINT should not be used without oversight by a physician knowledgeable in CPET and its interpretation. Moreover, knowledgeable physicians are encouraged to modify the XINT interpretations based on their own experience and ongoing advances in the field.

It should also be noted that the various "cut-points" chosen in the XINT program are necessarily somewhat arbitrary. Although we attempted to use clinically and physiologically meaningful values, unlike humans, computers require specific values. Under certain circumstances it is possible that the program could give misleading or inaccurate interpretations. However, by utilizing an integrative approach, this problem is minimized. Further, extensive testing has shown that most physiologic abnormalities are described by the XINT program and the interpretations are usually clinically meaningful and complete.

## Conclusion

In this paper we have discussed the logic underlying the XINT program and have shown its ability to render clinically useful interpretations consistent with ATS/ACCP criteria. The program and the open source code are available free at the XINT website [3]. It can also be downloaded from this article.

XINT is an example of medical interpretative software. We believe that this kind of software, that assists physicians in diagnosis and patient management, should be open source, peer-reviewed and published in medical journals like other important medical-related information. Undoubtedly, this type of software will be an integral part of medical care in the not-too-distant future.

## Availability and Requirements

Project Name: XINT

Project Home Page: 

Operating Systems: Microsoft Windows or Macintosh

Other Requirements: Active State Tcl (a)

License: None required

Restrictions to use: The interpretive software (XINT) and its documentation may be used for any purpose, provided that this notice is retained in all copies (or modified copies). No written agreement, license, or royalty fee is required for any of the authorized uses. Modifications of to this software are allowed. The software (or modified software) may not be sold or distributed for financial gain without the prior written authorization of Robert M Ross M.D. The ActiveState Tcl language is free to use but is covered by its own license agreement.

(a) ActiveState Tcl is copyrighted by the Regents of the University of California, Sun Microsystems, Inc., Scriptics Corporation, and other parties. Please see the license agreement on  for complete details.

Instructions for downloading XINT from this article:

In order to run the interpretation program, XINT, you must download the program and the programming language onto your computer.

Windows ^® ^Users:

To download the programs proceed as follows:

- Download "Additional File 5"(ActiveTcl8.4.4.0-win32-ix86.exe) to the desktop.

- Download the zip file, "Additional File 3" (CPX.zip) to the desktop.

- You have now downloaded the required files to run XINT.

- Extract the two files on your desktop by double-clicking on them. XINT is now ready to run.

^® ^Microsoft Redmond WA.

Running the XINT program in Windows:

- Open the CPX folder on your desktop.

- Double click on the "cpx.tcl" (or it may just show cpx without an extension).

- Information about the program and the licensing agreement can be found at the top left.

- The ATS/ACCP cardiopulmonary examples [1] can be found by pressing "load". Then highlight the case example and double click on it. The data will now be in the entry screen.

- To print or view the report press "Report". Note: the data must be in the entry screen to view or print a report.

- Under certain circumstances you may need to manually open the report with a text editor. The report file (cpxrpt.txt) is located in the CPX folder.

Macintosh ^® ^OSX users:

- Download "Additional File 6" (TclTkAquaBI-8.4.2.0.dmg) to the desktop. (Required for versions prior to 10.4 "Tiger")

- Download the zip file "Additional File 4" (CPXmac.zip) to the desktop.

- You have now downloaded the required files to run XINT.

- Double Click on the downloaded file to open the disk image, and then click on the file "TclTkAquaBI.mpkg" in the image to run the TCL installer.

- Double click on the downloaded CPXmac.zip on the desktop. Stuffit Expander will extract the files to a folder named CPXmac on the desktop.

- XINT is now ready to run.

^® ^Apple Inc. Cupertino CA

Running the XINT program in OSX:

- Open the CPXmac folder on your desktop.

- Double click on the application XINT_MACOS to start XINT.

- Information about the program and the licensing agreement can be found at the top left.

- The ATS/ACCP cardiopulmonary examples [1] can be found by pressing "load". Then highlight the case example and double click on it. The data will now be in the entry screen.

- To print or view the report press "Report". Note: the data must be in the entry screen to view or print a report.

- Under certain circumstances you may need to manually open the report with a text editor. The report file (cpxrpt.txt) is located in the CPX folder.

## Competing interests

The author(s) declare that they have no competing interests.

## Authors' contributions

DBC assisted in getting the software written, analyzing its usefulness and assisted in the creation of the manuscript.

RMR created the logic behind the XINT software. He also worked with the programmers in its development and its required modifications. He also analyzed its clinical usefulness and created the manuscript.

Both authors have read and approved the final manuscript.

## Pre-publication history

The pre-publication history for this paper can be accessed here:



## Supplementary Material

Additional file 1 XINTlogic. This file provides the detailed logic used by the XINT program. The variable names are described in Table 1. The actual source code may also be read directly simply by opening the source code with a text editor. Click here for file

Additional file 2 XINTinterpretations. These are the XINT generated reports based on the five examples provided in the ATS/ACCP statement on cardiopulmonary exercise testing [1]. Click here for file

Additional file 5 ActiveTcl8.4.4.0-win32-ix86. This is the file required for the language used by XINT to run in Windows. Click here for file

Additional file 3 CPX. These are the program files required to run the program in Windows. Click here for file

Additional file 6 TclTkAquaBI-8.4.2.0. This is the file required for the language used by XINT to run on a Mac. Click here for file

Additional file 4 CPXmac. These are the program files required to run the program on a Mac. Click here for file
