# Supplementary material for: Software for interpreting cardiopulmonary exercise tests
Source: BMC Pulm Med. 2007 Oct 23;7:15. doi: 10.1186/1471-2466-7-15 (PMC2121644; doi:10.1186/1471-2466-7-15)
Supplement: Additional file 1 — XINTlogic. This file provides the detailed logic used by the XINT program. The variable names are described in Table 1. The actual source code may also be read directly simply by opening the source code with a text editor. [file 1471-2466-7-15-S1.doc]

section a VO2max

“Cardiopulmonary Exercise Testing reveals a maximum oxygen uptake of __ ml/kg/min or __% of predicted.”

if ($pcntBf greater than 40 and $vo2MaxPp less than 80 ) then

set r1 [ expr $height / 39.37 ]

set maxVo2Kg [ expr round(($maxVo2Ml / (30.0 * $r1 * $r1)) * 10) / 10 ]

set vo2MaxPp [ expr round($maxVo2Kg * 100.0 / double($maxPVo2Kg)) ]

set atKg [ expr round(($atMl / (30.0 * $r1 * $r1)) * 10) / 10 ]

append tempPrn "However, based on a more normal BMI of 30, this would be about "

append tempPrn $maxVo2Kg

append tempPrn " ml/kg/min or "

append tempPrn $vo2MaxPp

append tempPrn "% of predicted. "

if ($vo2MaxPp greater than 120) then

append tempPrn $A1

set printA1 1

if (($vo2MaxPp greater than 80) and ($vo2MaxPp less than = 120)) then

append tempPrn $A2

if (not $Tt) then

append tempPrn $A7

else

append tempPrn $A6

if (($vo2MaxPp greater than 60) and ($vo2MaxPp less than = 80)) then

if ($maxVo2Kg greater than or equal to 20) then

append tempPrn $A3

else

append tempPrn $A11

if (not $Tt) then

append tempPrn $A7

else

append tempPrn $A5

if (($vo2MaxPp greater than 50) and ($vo2MaxPp less than = 60)) then

append tempPrn $A4

if (not $Tt) then

append tempPrn $A7

else

append tempPrn $A5

if ($vo2MaxPp less than = 50) then

append tempPrn $A13

if (not $Tt) then

append tempPrn $A7

else

append tempPrn $A5

if ([string length $due] not= 0 and not$printA1) then

append tempPrn $due

set A1 "This is high indicating good cardiorespiratory capacity. "

set A2 "This is within normal limits for an individual of this age and sex. "

set A3 "This is moderately reduced compatible with a mild functional aerobic impairment for an individual of this age and sex. "

set A4 "This is markedly reduced for an individual of this age and sex. "

set A5 "However, the test was terminated by the observer due to "

set A6 "The test was terminated by the observer due to "

set A7 "The patient stopped due to "

set A11 "This is significantly reduced for an individual of this age and sex. "

set A13 "This is severely reduced for an individual of this age and sex. "

section aa RPE

if $maxRPE not= 0 then

append tempPrn $A8

append tempPrn $maxRPE

append tempPrn "/10. "

if ( $maxRPE greater than 7) then

append tempPrn $A12

set A8 " The Maximum RPE achieved was "

set A12 "This suggests a maximal effort under appropriate clinical conditions. "

section ab VO2/External Power Output

if [string length $maxP] equals 0 then

if $maxPMph not= 0 then

if ( $maxVo2KgTmp / ($maxPMph * (262.5 + 17.5 * $maxPElev)/60)) greater than 1.2 then

if ( $vo2MaxPp greater than 70.0 ) then

append tempPrn $A10

else

append tempPrn $A10a

if ($maxVo2KgTmp / ($maxPMph * (262.5 + 17.5 * $maxPElev)/60)) less than 0.75 then

if ( $vo2MaxPp greater than 70.0 ) then

append tempPrn $A9

else

append tempPrn $A9a

else

if $maxP not= 0 then

if ((($maxP * 11.0) + 300.0) * 0.75) greater than or equal to ($maxVo2KgTmp * $weight * 0.454) then

if ( $vo2MaxPp greater than 70.0 ) then

append tempPrn $A9

else

append tempPrn $A9a

if ((($maxP * 11.0) + 300.0) * 1.2) less than ($maxVo2KgTmp * $weight * 0.454) then

if ( $vo2MaxPp greater than 70.0 ) then

append tempPrn $A10

else

append tempPrn $A10a

set A9 "The measured VO2 max is significantly less than predicted from the external power output. This suggests possible technical measurement problems. "

set A9a "The measured VO2 max is significantly less than predicted from the external power output. This may be due to abnormal oxygen kinetics or technical measurement problems. "

set A10 "The measured VO2 max is significantly greater than predicted from the external power output. This suggests the possibility of metabolic abnormalities or technical measurement problems. "

set A10a "The measured VO2 max is significantly greater than predicted from the external power output. This suggests possible technical measurement problems. "

section b RESTING HEART RATE

if ($restHr greater than 0) then

if ($restHr greater than or equal to 95) then

if ($vo2MaxPp greater than 80) then

append tempPrn $B1

else

append tempPrn $B2

elseif ($restHr less than = 60) then

if (not $bVe) then

if ($vo2MaxPp greater than 100) then

append tempPrn $B4

else

append tempPrn $B3

else

append tempPrn $B5

else

append tempPrn $B6

set B1 "The resting heart rate is elevated compatible with anxiety (or chronotropic drugs). "

set B2 "The resting heart rate is elevated. "

set B3 "The resting heart rate is low. "

set B4 "The resting heart rate is low compatible with good fitness and increased vagal tone. "

set B5 "The resting heart rate is low due to negative chronotropic agents or possible conduction abnormalities. "

set B6 "The resting heart rate is within normal limits. "

section c HEART RATE SLOPE

if ($hrVo2 not= 0) then

if ($pPHrVo2 greater than or equal to 80 and $pPHrVo2 less than = 120) then

append tempPrn $C1

if ($pPHrVo2 greater than 120 and $pPHrVo2 less than = 135) then

if ($vo2MaxPp greater than or equal to 100) then

append tempPrn $C4

elseif ($vo2MaxPp less than = 75) then

append tempPrn $C3

else

append tempPrn $C2

if ($pPHrVo2 greater than 135) then

append tempPrn $C3B

if ($pPHrVo2 less than 80) then

if ($vo2MaxPp greater than or equal to 100 ) then

append tempPrn $C5

else

append tempPrn $C7

set C1 "The heart rate response to exercise is within normal limits. "

set C2 "The heart rate response to exercise is high in keeping with mild unfitness. "

set C3 "The heart rate response to exercise is high. "

set C3B "The heart rate response to exercise is very high. "

set C4 ""

set C5 "The heart rate response to exercise is low indicative of good cardiovascular fitness and a large stroke volume. "

set C7 "The heart rate response to exercise is low. "

section d ANAEROBIC THRESHOLD

if ($atKg equals 0)then

set atneg 1

append tempPrn $D6

else

if ($vo2MaxPp greater than 80) then

append tempPrn $D1

if ($vo2MaxPp less than = 80 and $vo2MaxPp greater than or equal to 75) then

append tempPrn $D2

if ($vo2MaxPp less than 75 and $vo2MaxPp greater than or equal to 70 ) then

if ($atKg less than = (0.65 * $maxVo2Kg)) then

append tempPrn $D3

else

append tempPrn $D7

if ($vo2MaxPp less than 70 and $vo2MaxPp greater than or equal to 60 ) then

if ($atKg less than = (0.65 * $maxVo2Kg)) then

append tempPrn $D4

else

append tempPrn $D7

if ($vo2MaxPp less than 60 ) then

if ($atKg less than = (0.65 * $maxVo2Kg)) then

append tempPrn $D5

else

append tempPrn $D7

set atTemp [ expr int(((double($atKg) / double($maxVo2Kg)) * 100) + 0.5) ]

if ($vo2MaxPp greater than 120) then

if ($atKg greater than or equal to (0.65 * $maxVo2Kg)) then

append tempPrn "The anaerobic threshold occurs at "

append tempPrn $atTemp

append tempPrn "% VO2max indicating good cardiovascular conditioning. "

else

append tempPrn "The anaerobic threshold occurs at "

append tempPrn $atTemp

append tempPrn "% VO2max. "

set D1 "There is an anaerobic threshold which is indicative of a normal circulatory limitation to exercise. "

set D2 "There is an anaerobic threshold which is indicative of mild unfitness. "

set D3 "There is an anaerobic threshold which is indicative of mild cardiovascular pathology or significant unfitness. "

set D4 "There is an anaerobic threshold which is indicative of a significant cardiovascular limitation. "

set D5 "There is an anaerobic threshold which is indicative of significant cardiovascular pathology. "

set D6 "No clear-cut anaerobic threshold is noted. "

set D7 "There is evidence of an anaerobic threshold. "

section e Maximum heart rate

append tempPrn "The maximum heart rate achieved is "

append tempPrn " bpm or "

append tempPrn "% predicted. "

if ($pPMaxHr greater than 85) then

append tempPrn $E1

if ($pPMaxHr less than or equal to 85) then

if ($bVe) then

append tempPrn $E2

set E1 "This suggests a maximum cardiovascular effort. "

set E2 "However, this patient's heart rate response is expected to be low. "

section f BLOOD PRESSURE

if (not($restSBp equals 0 or $restDBp equals 0)) then

if ($restSBp greater than 160 or $restDBp greater than 105) then

append tempPrn $F2

set printedf2 1

elseif ($restSBp greater than 145 or $restDBp greater than 95) then

append tempPrn $F1

set printedf1 1

elseif ($restSBp less than 95 or $restDBp less than 60) then

append tempPrn $F3

set printedf3 1

elseif (($restSBp greater than or equal to 95 and $restSBp less than = 145) and

($restDBp greater than or equal to 60 and $restDBp less than = 95)) then

append tempPrn $F4

set printedf4 1

if ($bpfall equals "Yes") then

append tempPrn $F11

elseif ($exSBp equals 0 and $exDBp equals 0) then

append tempPrn ". "

elseif $printedf1 or $printedf2 then

if ($exDBp less than = 95 and \

$exSBp less than 220) then

append tempPrn $F6

else

append tempPrn $F7

elseif ($printedf4) then

if (($exDBp greater than 100 and ($exDBp greater than $restDBp + 5)) or \

$exSBp greater than 220) then

append tempPrn $F8

elseif ($exSBp less than 160 ) and ( $exSBp less than ($restSBp + 2.0 * $maxVo2Kg) ) then

append tempPrn $F9

else

append tempPrn $F7

elseif ($printedf3) then

if (($exDBp greater than 100 and ($exDBp greater than $restDBp + 5)) or \

$exSBp greater than 220) then

append tempPrn $F8

else

if ($exDBp greater than 70 or \

$exSBp greater than 140) then

append tempPrn $F6

else

append tempPrn $F7

elseif ($exSBp equals 0 or $exDBp equals 0) then

append tempPrn $F12

set F1 "Blood pressure is elevated at rest "

set F2 "Blood pressure is markedly elevated at rest "

set F3 "Blood pressure is low at rest "

set F4 "Blood pressure is normal at rest "

set F6 "but improves during exercise. "

set F7 "and during exercise. "

set F8 "but a hypertensive response is seen during exercise. "

set F9 "but does not rise adequately during exercise. "

set F11 ". Systolic blood pressure falls late in exercise. "

set F12 "No blood pressure data was obtained during exercise. "

section g EKG

if ($restEkg equals "not performed") then

append tempPrn $G10

elseif ($restEkg equals "abnormal") then

append tempPrn $G2

elseif ($restEkg equals "cor pulmonale") then

append tempPrn $G2B

else

append tempPrn $G1

set tempStr "The exercise EKG reveals "

set ekgAbnornal 0

if ($myoisc equals "Yes") then

append tempStr "positive EKG evidence for myocardial ischemia ("

append tempStr $mmSt

append tempStr " mm ST "

set ekgAbnornal 1

if ($stType equals "Elevation") then

append tempStr "elevation at a heart rate of "

else

append tempStr "depression at a heart rate of "

append tempStr " beats/min), "

if ($rhythm equals "Yes") then

set ekgAbnornal 1

append tempStr $G4

if ($conduct equals "Yes") then

set ekgAbnornal 1

append tempStr $G5

if ($minab equals "Yes") then

set ekgAbnornal 1

append tempStr $G6

if ($ekgAbnornal) then

append tempPrn [ string range $tempStr 0 [ expr [string length $tempStr] - 3 ] ]

append tempPrn ". "

elseif ($pvcs equals "Yes") then

append tempPrn "The exercise EKG reveals "

append tempPrn $G7

append tempPrn ". "

elseif ($exEkg equals "normal") then

append tempPrn "The exercise EKG reveals "

append tempPrn $G8

append tempPrn ". "

if ($frpt equals "Yes") then

append tempPrn "Please see formal EKG report. "

set G1 "The EKG is normal at rest. "

set G2 "The resting EKG is abnormal. "

set G2B "The resting EKG suggests Cor Pulmonale. "

set G4 "significant rhythm disturbances"

set G5 "significant cardiac conduction abnormalities"

set G6 "non-specific changes"

set G7 "PVC's which were initially present reduced, suggesting a benign etiology"

set G8 "no diagnostic abnormalities"

set G9 "A diagnostic EKG was not performed. "

set G10 "An EKG was not performed during this test. "

section h RESTING SPIROMETRY

if (not($preFvc equals 0)) then

if ($preFev1Fvc less than 62) then

append tempPrn $H8

else

if ($preFev1Fvc less than 70) then

if ($prePPFvc greater than or equal to 75) then

append tempPrn $H5

else

append tempPrn $H3

elseif ($prePPFvc less than 75 and $preFev1Fvc greater than or equal to 73) then

append tempPrn $H2

elseif ($prePPFvc less than 75 and $preFev1Fvc less than 73) then

append tempPrn $H7

elseif ($prePPFvc greater than 85 and $preFev1Fvc greater than or equal to 73) then

append tempPrn $H4

else

append tempPrn $H6

set H2 "Resting spirometry reveals restriction. "

set H3 "Resting spirometry reveals restriction and obstruction. "

set H4 "Resting spirometry is normal. "

set H5 "Resting spirometry reveals airflow obstruction. "

set H6 "Resting spirometry does not reveal any functionally significant abnormalities. "

set H7 "Resting spirometry reveals restriction with superimposed mild airflow obstruction. "

set H8 "Resting spirometry reveals significant airflow obstruction. "

section i POST EXERCISE SPIROMETRY

if ($postFvc equals 0) then

append tempPrn $I5

else

set rf [ expr round($postFev1Fvc) ]

set ri [ expr round($preFev1Fvc) ]

if ($rf less than = ($ri - 4) and $ri greater than or equal to 72 and $rf less than = 72) then

append tempPrn $I1

elseif ($rf less than = ($ri - 4) and $ri less than 72) then

append tempPrn $I2

else

append tempPrn $I4

set I1 "There is evidence of exercise induced asthma. "

set I2 "Airflow obstruction worsens after exercise. "

set I4 "Exercise does not induce a significant asthmatic response. "

set I5 "No post exercise spirometry was performed. "

section j VENTILATION

if ($restVe greater than or equal to 12) then

append tempPrn $JA1

if ($midVeVo2 not= 0) then

if ($midVeVo2 greater than 32) then

append tempPrn $JB1

if ($midVeVo2 less than = 32 and \

$midVeVo2 greater than 22) then

append tempPrn $JB2

if ($midVeVo2 less than = 22) then

append tempPrn $JB3

if ($vo2MaxPp less than 50) then

if ($maxPPVe greater than or equal to 80) then

append tempPrn $JC1

else

append tempPrn $JC2

elseif ($vo2MaxPp greater than or equal to 50 and $vo2MaxPp less than = 80) then

if ($maxPPVe greater than or equal to 95) then

append tempPrn $JC3

if ($maxPPVe greater than or equal to 85 and \

$maxPPVe less than 95) then

append tempPrn $JC4

if ($maxPPVe less than 85) then

append tempPrn $JC6

elseif ($vo2MaxPp greater than 80 and $vo2MaxPp less than = 120) then

if ($maxPPVe greater than or equal to 95) then

append tempPrn $JC7

if ($maxPPVe greater than or equal to 85 and \

$maxPPVe less than 95) then

append tempPrn $JC4

if ($maxPPVe less than 85) then

append tempPrn $JC6

set JA1 "Ventilation is elevated at rest. "

set JB1 "There is a high ventilatory response to exercise. "

set JB2 "There is a normal ventilatory response to exercise. "

set JB3 "there is a low ventilatory response to exercise. "

set JC1 "There is a marked reduction in exercise capacity with a significant ventilatory limitation. "

set JC2 "A ventilatory limitation to exercise is not seen. "

set JC3 "Maximum predicted ventilation is achieved indicating a ventilatory limitation to exercise. "

set JC4 "Ventilatory reserve is significantly diminished. "

set JC6 "Maximum predicted ventilation is not achieved. "

set JC7 "Maximum predicted ventilation is achieved suggesting a ventilatory limitation to exercise. "

section k BREATHING PATTERN TIDAL VOLUME

if ($vo2MaxPp less than 55) then

if ($prePPFvc less than 70) then

if ($maxTv less than (0.5 * $preFvc)) then

append tempPrn $K5

else

append tempPrn $K4

elseif ($maxTv less than (0.35 * $preFvc)) then

append tempPrn $K7

else

append tempPrn $K4

elseif ($prePPFvc less than 70) then

if ($maxTv less than (0.5 * $preFvc)) then

append tempPrn $K5

else

append tempPrn $K2

elseif ($maxTv less than (0.45 * $preFvc)) then

append tempPrn $K3

else

append tempPrn $K4

set K2 "The respiratory frequency is elevated compatible with the reduced vital capacity. "

set K3 "There is evidence of shallow breathing. "

set K4 "No major abnormality of breathing pattern is noted. "

set K5 "The breathing pattern is rapid. "

set K7 "The respiratory pattern suggests shallow breathing. "

section l BLOOD GASES ACID-BASE

if ($AbgRoom) then

if ($h less than = 48) then

if ($h less than 32) then

append tempPrn $L1

if ($rraHco3 greater than or equal to 27 and $paCo2Room less than = 35) then

append tempPrn $L6

elseif ($rraHco3 greater than or equal to 27 and $paCo2Room greater than 35) then

append tempPrn $L5

elseif ($rraHco3 less than 27 and $paCo2Room less than = 35) then

append tempPrn $L4

else

append tempPrn $L7

else

append tempPrn $L2

if ($paCo2Room less than 35) then

append tempPrn $L12

if ($paCo2Room greater than 45) then

append tempPrn $L13

else

append tempPrn $L3

if ($rraHco3 less than 24 and $paCo2Room greater than 42) then

append tempPrn $L10

elseif ($hco3 less than 24 and $paCo2Room less than = 42) then

append tempPrn $L9

elseif ($hco3 greater than or equal to 24 and $paCo2Room greater than 42) then

append tempPrn $L8

else

append tempPrn $L7

set L1 "Acid base status at rest reveals alkalosis "

set L2 "Acid base status at rest is normal. "

set L3 "Acid base status at rest reveals acidosis "

set L4 "primarily due to a respiratory component. "

set L5 "primarily due to a metabolic component. "

set L6 "with both a respiratory and metabolic component. "

set L7 "which is mild. "

set L8 "primarily due to a respiratory component. "

set L9 "primarily due to a metabolic component. "

set L10 "due to both a respiratory and metabolic component. "

set L12 "However, there is both a respiratory alkalosis and a metabolic acidosis present. "

set L13 "However, there is both a respiratory acidosis and a metabolic alkalosis present. "

section m ACID BASE - AFTER EXERCISE

if ($abgExer) then

if ($exHco3 less than = ($rraHco3 - 3.5)) then

append tempPrn $M1

set test1 1

if ($exHco3 greater than ($rraHco3 - 2)) then

append tempPrn $M2

set test2 1

if (not$test1 and not$test2 and $exPaCo2 less than = ($rraPacO2 - 4)) then

append tempPrn $M3

set M1 "After exercise there is evidence of lactic acidosis. "

set M2 "There is no significant lactic acidosis after exercise. "

set M3 "After exercise there is evidence of mild lactic acidosis. "

section n ALVEOLAR VENTILATION AND GAS EXCHANGE

if ($paCo2 less than = 50 and $paCo2 greater than or equal to 45) then

append tempPrn $N1

elseif ($paCo2 greater than 50) then

append tempPrn $N2

elseif ($paCo2 less than = 35) then

append tempPrn $N3

else

append tempPrn $N4

if ($ao2 greater than 25 and $po2 less than 60) then

append tempPrn $N6

elseif ($ao2 greater than 25 and $po2 less than 85) then

append tempPrn $N5

elseif ($ao2 less than = 25 and $ao2 greater than or equal to 20 and $po2 less than 85) then

append tempPrn $N75

else

append tempPrn $N7

set N1 "moderate alveolar hypoventilation "

set N2 "severe alveolar hypoventilation "

set N3 "alveolar hyperventilation "

set N4 "normal alveolar ventilation "

set N5 "with hypoxemia. "

set N6 "with significant hypoxemia. "

set N7 "with normal oxygenation. "

set N75 "with mild hypoxemia. "

section q ALVEOLAR VENTILATION at end of exercise

if ($rraPacO2 less than = 45) then

if ($rraPacO2 greater than 35) then

if ($exPaCo2 greater than or equal to ($rraPacO2 + 4 ) and $exPaCo2 greater than 45) then

append tempPrn $Q8

elseif ($exPaCo2 less than ($rraPacO2 - 3) and $exPaCo2 less than 35) then

append tempPrn $Q9

else

append tempPrn $Q10

elseif ($exPaCo2 greater than 43) then

append tempPrn $Q8

elseif ($exPaCo2 greater than 37) then

append tempPrn $Q4

elseif ($exPaCo2 greater than or equal to ($rraPacO2 + 4)) then

append tempPrn $Q5

elseif ($exPaCo2 less than = ($rraPacO2 - 4)) then

append tempPrn $Q11

else

append tempPrn $Q7

else

if ($exPaCo2 greater than or equal to ($rraPacO2 + 3)) then

append tempPrn $Q1

if ($exPaCo2 greater than or equal to ($rraPacO2 - 3) and $exPaCo2 less than ($rraPacO2 + 3)) then

append tempPrn $Q2

if ($exPaCo2 less than ($rraPacO2 - 3)) then

if ($exPaCo2 less than 44) then

append tempPrn $Q4

else

append tempPrn $Q3

set Q1 "Alveolar ventilation worsens with exercise. "

set Q2 "There is no significant change in alveolar ventilation with exercise. "

set Q3 "Alveolar ventilation improves somewhat with exercise. "

set Q4 "Alveolar ventilation becomes normal with exercise. "

set Q5 "Alveolar hyperventilation improves somewhat with exercise. "

set Q7 "There is no significant change in alveolar hyperventilation with exercise. "

set Q8 "After exercise alveolar hypoventilation becomes manifest. "

set Q9 "Alveolar hyperventilation occurs with exercise. "

set Q10 "There is no significant abnormality of alveolar ventilation with exercise. "

set Q11 "Alveolar hyperventilation increases further with exercise. "

section r GAS EXCHANGE WITH EXERCISE

if ($abgExer and ($AbgRoom or $AbgOxy)) then

if ($aai less than = 20 or $po2r greater than or equal to 80) then

set aarest 1

if ( $maxVe greater than or equal to 20) then

set veok 1

if ($maxVdVt greater than or equal to 0.3) then

set vdt 1

if ($aaf greater than or equal to ($aai + 10) and $exPaO2 less than = 60) then

set aaw 1

elseif (($aaf greater than ($aai - 5) and $aaf less than ($aai + 10)) or \

($aaf greater than or equal to ($aai + 10) and $exPaO2 greater than 60)) then

set aan 1

elseif ($aaf less than = ($aai - 5)) then

set aab 1

if $aarest then

if $veok then

if ($aaf greater than or equal to 30 and $exPaO2 less than 80 and $vdt) then

append tempPrn $R13

elseif ($aaf greater than or equal to 30 and $exPaO2 less than 80) then

append tempPrn $R14

elseif $vdt then

append tempPrn $R15

else

append tempPrn $R16

else

if ($aaf greater than or equal to 30 and $exPaO2 less than 80) then

append tempPrn $R17

append tempPrn $RA

else

append tempPrn $R16

append tempPrn $RA1

if not $aarest then

if $aaw then

if $veok then

if $vdt then

append tempPrn $R1

else

append tempPrn $R2

else

append tempPrn $R2A

append tempPrn $RA

if $aan then

if ( $veok ) then

if $vdt then

append tempPrn $R3

else

append tempPrn $R10

else

append tempPrn $R10

append tempPrn $RA1

if $aab then

if ( $veok ) then

if $vdt then

if ($aaf less than 25 or $exPaO2 greater than 80) then

append tempPrn $R6

else

append tempPrn $R5

else

if ($aaf less than 25 or $exPaO2 greater than 80) then

append tempPrn $R8

else

append tempPrn $R7

else

if ($aaf less than 25 or $exPaO2 greater than 80) then

append tempPrn $R8

append tempPrn $RA1

else

append tempPrn $R7

append tempPrn $RA1

set R1 "There is worsening of gas exchange as evidenced by a widening of the alveolar-arterial oxygen gradient and high dead space ventilation. "

set R2 "There is worsening of gas exchange as evidenced by a widened alveolar-arterial oxygen gradient but dead space ventilation (areas of high V\/Q) remained relatively normal. "

set R2A "There is worsening of gas exchange as evidenced by a widened alveolar-arterial oxygen gradient."

set R3 "Dead space ventilation (areas of high V\/Q) is high, indicating wasted ventilation. \"Shunt\" fraction (areas of low V\/Q) however remains relatively unchanged. "

set R5 "Gas exchange improves somewhat in areas of low V\/Q (shunt). However, dead space ventilation (areas of high V\/Q)is high, indicating wasted ventilation. "

set R6 "Gas exchange improves to normal in areas of low V\/Q (shunt). However, dead space ventilation (areas of high V\/Q)is elevated, indicating wasted ventilation. "

set R7 "Gas exchange improves somewhat. "

set R8 "Gas exchange improves to normal. "

set R10 "Gas exchange remains relatively unchanged. "

set R13 "Gas exchange becomes abnormal with increased areas of both high and low V\/Q (\"dead space\" and \"shunt\" fraction) leading to wasted ventilation and pulmonary circulation. "

set R14 "Gas exchange becomes abnormal with increased areas of low V\/Q (\"shunt\" fraction) leading to wasted pulmonary circulation. Dead space ventilation remains normal. "

set R15 "There is increased \"dead space\" ventilation, otherwise gas exchange is normal. "

set R16 "There is no significant abnormality of gas exchange. "

set R17 "Gas exchange becomes abnormal with increased areas of low V\/Q (\"shunt\" fraction). "

set RA "(Vd\/Vt cannot be accurately assessed at these low ventilations) "

set RA1 "(However, Vd\/Vt cannot be accurately assessed at these low ventilations) "

section s

if ($rraO2Sat not= 0) then

set satE $rraO2Sat

set tempStr "room air "

if ($satE greater than 85 and $satE less than 90) then

append tempPrn "At rest on "

append tempPrn $tempStr

append tempPrn "there is moderate oxygen desaturation. "

elseif ($satE less than = 85 and $satE greater than 75) then

append tempPrn "At rest on "

append tempPrn $tempStr

append tempPrn "there is marked oxygen desaturation. "

elseif ($satE less than = 75) then

append tempPrn "At rest on "

append tempPrn $tempStr

append tempPrn "there is severe oxygen desaturation. "

else

append tempPrn "At rest on "

append tempPrn $tempStr

append tempPrn "the oxygen saturation is adequate. "

section u EXERCISE O2 SAT

if ($exO2Sat not= 0) then

if ($rraO2Sat not= 0 ) then

set satr $rraO2Sat

if ($satr greater than or equal to 90) then

if ($exO2Sat greater than or equal to 90 and $exO2Sat less than = ($satr - 5)) then

append tempPrn $U5B

elseif ($exO2Sat greater than or equal to 85 and $exO2Sat less than 90 and

$exO2Sat less than = ($satr - 4)) then

append tempPrn $U5

elseif ($exO2Sat less than 85) then

append tempPrn $U6

else

append tempPrn $U7

else

if ($exO2Sat less than = ($satr - 4)) then

append tempPrn $U1

else

if ($exO2Sat less than ($satr + 2) and \

$exO2Sat greater than ($satr - 4)) then

if ($exO2Sat less than 90) then

append tempPrn $U2

set endU 1

if ( not $endU) then

append tempPrn $U3

if ($exO2Sat greater than or equal to ($satr + 2)) and (not $endU) then

if ($exO2Sat less than 90) and (not $endU) then

append tempPrn $U4

elseif (not $endU) then

append tempPrn $U3

set endU 1

if ($exO2Sat greater than 85) and ($exO2Sat less than 90) and (not $endU) then

append tempPrn $U8

elseif ($exO2Sat greater than or equal to 75) and ($exO2Sat less than = 85) and (not $endU) then

append tempPrn $U9

elseif ($exO2Sat less than 75) and (not $endU) then

append tempPrn $U10

elseif (not $endU) then

append tempPrn $U11

set U1 "During exercise there is further oxygen desaturation. "

set U2 "During exercise oxygen saturation does not change significantly. "

set U3 "During exercise oxygen saturation improves to normal. "

set U4 "During exercise oxygen saturation improves somewhat. "

set U5B "During exercise there is mild arterial desaturation. "

set U5 "During exercise there is significant arterial desaturation. "

set U6 "During exercise there is marked arterial desaturation. "

set U7 "During exercise there is no significant arterial desaturation. "

set U8 "During exercise there is moderate oxygen desaturation. "

set U9 "During exercise there is marked oxygen desaturation. "

set U10 "During exercise there is severe oxygen desaturation. "

set U11 "During exercise oxygen saturation is adequate. "

section v CONCLUSION

CONCLUSION:

“Exercise performance "

if ($vo2MaxPp greater than or equal to 110) then

if $pcntBf greater than 40 and ($vo2MaxPpTmp less than 80) then

set tempStr $V1a

else

set tempStr $V1

if ($vo2MaxPp less than 110) then

if $pcntBf greater than 40 and ($vo2MaxPpTmp less than 80) then

set tempStr $V2a

else

set tempStr $V2

if ($vo2MaxPp less than 80) then

if $pcntBf greater than 40 then

set tempStr $V3a

else

set tempStr $V3

if ($vo2MaxPp less than 70) then

if $pcntBf greater than 40 then

set tempStr $V4a

else

set tempStr $V4

if ($vo2MaxPp less than 55) then

if ($maxVo2Kg greater than or equal to 20 ) then

if $pcntBf greater than 40 then

set tempStr $V5a

else

set tempStr $V5

else

if $pcntBf greater than 40 then

set tempStr $V6a

else

set tempStr $V6

append tempPrn $tempStr

if ($Tt) then

if ($vo2MaxPp greater than or equal to 85) then

append tempPrn $W1

append tempPrn $due

else

append tempPrn $W2

append tempPrn $due

elseif ($vo2MaxPp less than 80) then

append tempPrn $W4

append tempPrn $due

set V1 "is excellent. "

set V2 "is normal. "

set V3 "is mildly reduced. "

set V4 "is significantly reduced. "

set V5 "is markedly reduced. "

set V6 "is severely reduced. "

set V1a ", based on a more normal body weight, is excellent. "

set V2a ", based on a more normal body weight, is normal. "

set V3a ", based even on a more normal body weight, is mildly reduced. "

set V4a ", based even on a more normal body weight, is significantly reduced. "

set V5a ", based even on a more normal body weight, is markedly reduced. "

set V6a ", based even on a more normal body weight, is severely reduced. "

set W1 "\n- The test was terminated by the observer due to "

set W2 "\n- However, the test was terminated by the observer due to "

set W4 "\n- The patient stopped due to "

section x1

if ($rf less than = ($ri - 4) and $ri greater than or equal to 72 and $rf less than = 72 and $postFev1Fvc not= 0) then

append tempPrn $X1

set X1 "\n- Exercise induced asthma. Bronchodilator treatment may improve exercise tolerance. "

section x KEY POINTS OBSTRUCTIVE

set printX 0

if (not$abgExer and $exO2Sat equals 0 and $paCo2Exer equals 0) then

if ($preFev1Fvc less than = 68 and $prePPFev1 less than = 50) then

append tempPrn $X8

set printX 1

elseif ($preFev1Fvc less than = 68 and \

$midVeVo2 greater than 35 and \

$vo2MaxPp less than = 70 and not$atKg) then

append tempPrn $X9

set printX 1

elseif ($preFev1Fvc less than = 68) then

append tempPrn $X2

set printX 1

else

if ($preFev1Fvc less than = 68 and $prePPFev1 less than = 50) then

if ($abgExer) then

set xxx [ expr ($exO2 * $baroPress * 0.01) - \

(double($paCo2Exer) / 0.8) - double($exPaO2) ]

else

set xxx 0

if ((30 less than = $xxx or \

($exO2Sat less than = ($satr - 4) and $exO2Sat less than 90) ) and \

$exO2Sat not= 0 and $paCo2Exer greater than or equal to 45) then

set printX 1

append tempPrn $X3

if (not$printX) then

if ($preFev1Fvc less than = 68 and $prePPFev1 less than = 50) then

if ($paCo2Exer greater than 45) then

append tempPrn $X4

set printX 1

if (not$printX) then

if ($preFev1Fvc less than = 68 and $prePPFev1 less than = 50) then

if ($abgExer) then

set xxx [ expr ($exO2 * $baroPress * 0.01) - \

(double($paCo2Exer) / 0.8) - double($exPaO2) ]

else

set xxx 0

if (30 less than = $xxx or $exO2Sat less than = ($satr - 4) and \

($exO2Sat less than 90 and $exO2Sat not= 0)) then

append tempPrn $X5

set printX 1

if (not$printX) then

if ($preFev1Fvc less than = 68 and $prePPFev1 less than = 50) then

append tempPrn $X8

set printX 1

elseif ($preFev1Fvc less than = 68 and $prePPFev1 greater than 50) then

append tempPrn $X2

set printX 1

set X2 "\n- Obstructive airways disease. Bronchodilator treatment may improve exercise tolerance. "

set X3 "\n- Severe obstructive airways disease with alveolar hypoventilation and hypoxemia. Supplemental oxygen during exercise may improve exercise tolerance. "

set X4 "\n- Severe obstructive airways disease with alveolar hypoventilation. Sedation should be given only with caution. Bronchodilator treatment may improve exercise tolerance. "

set X5 "\n- Severe obstructive airways disease with hypoxemia. Supplemental oxygen during exercise may improve exercise tolerance. "

set X7 "\n- There is evidence of inappropriate hyperventilation. Exercise training and\/or pharmacotherapy may improve exercise tolerance and\/or reduce the sensation of dyspnea. "

set X8 "\n- Severe obstructive airways disease. Bronchodilators and\/or supplemental oxygen may improve exercise tolerance. "

set X9 "\n- Wasted ventilation during exercise. This may be due to gas exchange abnormalities, and\/or anxiety. Further studies with blood gases may be appropriate if clinically indicated. "

section y

set printY 0

if $restEkg equals "not performed" then

append tempPrn $Y1

elseif $restEkg equals "cor pulmonale" then

append tempPrn $Y1B

if ($myoisc equals "Yes")

if ($rhythm equals "Yes")

if ($conduct equals "Yes")

if ($minab equals "Yes")

if ($bpfall equals "Yes")

set Temp1 "\n- Exercise induced "

append tempStr "myocardial ischemia. "

append tempStr "morbid arrythmias. "

append tempStr "significant conduction abnormalities. "

append tempStr "minor EKG abnormalities. "

append tempStr "exercise hypotension. "

set Temp2 "Appropriate further evaluation and treatment is suggested if clinically indicated. "

if ($pvcs equals "Yes") and the above items not printed then

append tempPrn $Y3

set Y1 "\n- No diagnostic EKG was performed. "

set Y1B "\n- EKG evidence of Cor Pulmonale. "

set Y3 "\n- probable benign PVC's. "

section low stroke volume

set Hr 0

set th 0

set hc 0

set hv 0

set CVtestLow 0

set printZ 0

if ($pPMaxHr greater than or equal to 85) then

incr Hr

if ($atKg not= 0 and ( ($atKg less than = (0.65 * $maxVo2Kg)) or (2 * $atKg less than 0.75 * $maxPVo2Kg) )) then

incr th

if ( $exHco3 not= 0 ) and ($exHco3 less than ($hco3r - 2)) then

incr hc

if ($pPHrVo2 greater than 120) then

incr hv

if ($th) then

set hc 0

if $bVe equals 1 then

if (($Hr + $th + $hc + $hv) greater than or equal to 1) then

set CVtestLow 1

else

if (($Hr + $th + $hc + $hv) greater than or equal to 2) then

set CVtestLow 1

section aa

if (($abgExer and $AbgRoom) or ($exO2Sat not= 0 and $satrnot= 0 )) then

if ($preFev1Fvc greater than 68 and $vo2MaxPp less than = 70) then

if ((( ($exO2Sat less than = ($satr - 4) and $exO2Sat less than 90) and \

($exO2Sat not= 0 and $satrnot= 0)) ) and \

($CVtestLow)) then

append tempPrn $CLA1

set ignorez 1

set printAa 1

set endAa 1

elseif (($maxVdVt greater than or equal to 0.3 and $maxVe greater than 20) and \

($CVtestLow)) then

append tempPrn $CLA1A

set ignorez 1

set printAa 1

set endAa 1

elseif ((($exO2Sat less than = ($satr - 4) and $exO2Sat less than 90)) or \

($maxVdVt greater than or equal to 0.3 and $maxVe greater than 20)) then

append tempPrn $CLA2

set printAa 1

set endAa 1

elseif ($maxVdVt greater than or equal to 0.3 and $maxVe greater than 20) then

append tempPrn $CLA2A

set printAa 1

set endAa 1

elseif ($preFev1Fvc greater than 68 and $vo2MaxPp greater than 70) then

if ((($exO2Sat less than = ($satr - 4) and $exO2Sat less than 90)) or \

($maxVdVt greater than or equal to 0.3 and $maxVe greater than 20)) then

append tempPrn $CLA2B

set printAa 1

set printCLA2A 1

set endAa 1

if (not$abgExer and $exO2Sat equals 0 and $paCo2Exer equals 0) then

if ($preFev1Fvc greater than 68 and $vo2MaxPp less than = 70) then

if ($maxVe greater than 20 and $midVeVo2 greater than 35) then

if ($CVtestLow) and \

( not $endAa ) then

append tempPrn $CLA5

set printAa 1

if $printCLA2A then set printAa 0

section vo2/watts

if ($wattsPPVo2 not= 0 ) then

if ($vo2MaxPp less than 70) and ($maxVo2Kg less than 20 ) then

if ($wattsPPVo2 less than 75) then

append tempPrn $CLB2

set printWatt 1

if ($wattsPPVo2 greater than 120) then

append tempPrn $CLB1

set printWatt 1

set CLB1 "\n- There is a high oxygen cost relative to the external power output achieved. This suggests the possibility of metabolic abnormalities contributing to reduced exercise capacity. "

set CLB2 "\n- There is a low oxygen cost relative to the external power output. This suggests abnormalities of oxygen transport mechanisms. "

section z

if not $ignorez then

if ($CVtestLow and $vo2MaxPp greater than or equal to 65 and $vo2MaxPp less than 85) then

append tempPrn $Z1

set printZ 1

if ($CVtestLow and $vo2MaxPp less than 65) then

append tempPrn $Z2

set printZ 1

else

if (($atKg not= 0 and (($atKg less than = (0.65 * $maxVo2Kg )) or (2 * $atKg less than 0.75 * $maxPVo2Kg) ) or \

($exHco3 less than ($hco3r - 2))) and ($wattsPPVo2 less than 75) and ($wattsPPVo2 greater than 0) and ($vo2MaxPp less than 60)) then

append tempPrn $Z3

set printZ 1

if ( not$printZ ) then

set CVtestLow 0

if (($th + $hc) greater than or equal to 1) then

set CVtestLow 1

if ($CVtestLow and $vo2MaxPp greater than or equal to 65 and $vo2MaxPp less than 85) then

append tempPrn $Z4

set printZ 1

if ($CVtestLow and $vo2MaxPp less than 65) then

append tempPrn $Z5

set printZ 1

section aa continued (This part only if nothing in “Z” is printed):

if not($printZ) then

if ($abgExer) then

if (($midVeVo2 greater than 35) and \

($preFev1Fvc greater than 68) and \

( not $CVtestLow)and \

($maxPPVe less than 80) and \

($vo2MaxPp less than = 70) and \

( not $endAa ) and \

($paCo2Exer less than = 35) and ($paCo2Exer not= 0) and (not$Tt)) then

append tempPrn $CLA4

set printAa 1

set endAa 1

if (($midVeVo2 greater than 35) and \

($preFev1Fvc greater than 68) and \

( not $CVtestLow) and \

($vo2MaxPp less than = 70) and \

( not $endAa ) and \

($paCo2Exer less than = 35) and ($paCo2Exer not= 0) and (not$Tt)) then

append tempPrn $CLA4

set printAa 1

set endAa 1

if (not$abgExer) then

if (($midVeVo2 greater than 35) and \

($preFev1Fvc greater than 68) and \

( not $CVtestLow) and \

($vo2MaxPp less than = 70) and \

( not $endAa ) and (not$Tt)) then

append tempPrn $CLA4A

set printAa 1

set endAa 1

if (not$abgExer and $exO2Sat equals 0 and $paCo2Exer equals 0) then

if ($preFev1Fvc greater than 68 and $vo2MaxPp less than = 70) then

if ($maxVe greater than 20 and $midVeVo2 greater than 35) then

if (not$CVtestLow) and \

( not $endAa ) then

append tempPrn $CLA6

set printAa 1

set Z1 "\n- Reduced stroke volume compatible with unfitness or mild functional cardiac impairment. Aerobic training may improve exercise tolerance if there is no evidence of significant valvular or primary myocardial disease. "

set Z2 "\n- Reduced cardiac capacity for exercise as seen in diseases of the left or right heart leading to a low stroke volume. Further evaluation is suggested if clinically indicated. "

set Z3 "\n- Possible acute cardiac failure. "

set Z4 "\n- Possible reduced stroke volume compatible with unfitness or mild functional cardiac impairment. Aerobic training may improve exercise tolerance if there is no evidence of significant valvular or primary myocardial disease. "

set Z5 "\n- Probable reduced cardiac capacity for exercise as seen in diseases of the left or right heart leading to a low stroke volume. Further evaluation is suggested if clinically indicated. "

set CLA1 "\n- Cardiopulmonary disorder leading to a low stroke volume and gas exchange abnormalities. Further studies to evaluate the possibility of interstitial\/alveolar lung disease and\/or pulmonary vascular disease are suggested if indicated. "

set CLA1A "\n- Cardiopulmonary disorder leading to a low stroke volume and wasted ventilation. Further studies to evaluate the possibility of pulmonary vascular disease or CHF are suggested if clinically indicated. "

set CLA2 "\n- Non-obstructive pulmonary disorder leading to gas exchange abnormalities. Further studies to evaluate the possibility of interstitial\/alveolar lung disease are suggested if clinically indicated. "

set CLA2A "\n- Non-obstructive pulmonary disorder leading to wasted ventilation. Further studies to evaluate the possibility of pulmonary vascular disease or CHF are suggested if clinically indicated. "

set CLA2B "\n- Evidence of gas exchange abnormalities. "

set CLA4 "\n- Alveolar hyperventilation without evidence of significant gas exchange abnormalities or lactic acidosis. This suggests anxiety or disordered breathing. Exercise training and\/or mild respiratory depressants might improve exercise capacity or the sensation dyspnea. "

set CLA4A "\n- Although gas exchange abnormalities cannot be excluded, the findings suggest hyperventilation due to anxiety or disordered breathing. Further studies with blood gases may be appropriate if clinically indicated. "

set CLA5 "\n- Possibly significant cardiopulmonary disorder which is leading to a high ventilatory response to exercise. Suggest further evaluation such as exercise with blood gases if clinically indicated. "

set CLA6 "\n- Wasted ventilation during exercise which may be leading to dyspnea and reduced exercise tolerance. Suggest further evaluation such as exercise with blood gases if clinically indicated. "

section bb

if ($maxVe greater than or equal to (0.95 * $maxPVe) and $vo2MaxPp less than 80) then

append tempPrn $BB1

set printBb 1

set BB1 "\n- There is a ventilatory limitation to exercise. This is abnormal. "

section cc

if (not($printX or $printY or $printZ or $printAa or $printBb or $printWatt )) then

if ($vo2MaxPp greater than or equal to 85) then

if ($atKg greater than 0) then

incr ctest

if ($pPMaxHr greater than or equal to 85) then

incr ctest

if ($exHco3 less than ($hco3r - 2)) then

incr ctest

if ($ctest greater than or equal to 2) then

append tempPrn $CC1

else

append tempPrn $CC2

set CC1 "\n- Functionally normal exercise evaluation. "

set CC2 "\n- There is no obvious cardiovascular or pulmonary exercise limitation on this evaluation. "

section METs

if ($vo2MaxPp less than 80) then

if (($atKg not= 0 and ($atKg less than = (0.65 * $maxVo2Kg))) or ($exHco3 less than ($hco3r - 3))) then

set mets1 [ expr double($atKg) / 3.5 ]

else

set mets1 0

if ($maxVe greater than (0.9 * $maxPVe)) then

set mets2 [ expr 0.5 * (double($maxVo2Kg) / 3.5) ]

else

set mets2 0

if ($mets1 not= 0 and $mets2 not= 0) then

if $mets1 less than $mets2 then

set mets $mets1

else

set mets $mets2

elseif ($mets1 not= 0) then

set mets $mets1

elseif ($mets2 not= 0) then

set mets $mets2

else

set mets 0

if ($mets equals 0) then

set testCc3Flag 0

else

set tmets [ expr round($mets * 2.0) ]

set mets [ expr $tmets / 2.0 ]

if ($mets less than 1.0) then

set mets 1.0

set testCc3Flag 1

set cc3 "\nThis patient's 8 hour work capacity is "

append cc3 $mets

if ($mets equals 1) then

append cc3 " MET."

else

append cc3 " METs."

append cc3 " Energy requirements are approximately 2.5-3 METs "

append cc3 "for office work, 3-4 METs for domestic or light "

append cc3 "factory work, 4-6 METs for outdoor or regular "

append cc3 "factory work, and above 6 METs for heavy work. "

append tempPrn $cc3
