# Supplementary material for: Software for interpreting cardiopulmonary exercise tests
Source: BMC Pulm Med. 2007 Oct 23;7:15. doi: 10.1186/1471-2466-7-15 (PMC2121644; doi:10.1186/1471-2466-7-15)
Supplement: Additional file 2 — XINTinterpretations. These are the XINT generated reports based on the five examples provided in the ATS/ACCP statement on cardiopulmonary exercise testing [1]. [file 1471-2466-7-15-S2.doc]

| Example One  Diagnosis: (Normal)    Age: 62 Gender: Male Height (in) : 69.0 (cm): 175.3  BMI: 27.3 Weight (lbs): 185.0 (kg): 84.0  Exercise Time: 9:0 Barometric Pressure (mm Hg): 722  RESULTS  SPIROMETRY ACTUAL PREDICTED %PREDICTED  Pre Exercise  FEV1 (L BTPS) 3.10 3.50 89  FVC (L BTPS) 4.50 4.55 99  ratio (%) 69 77 90  MVV (L BTPS) 124 123 101    CARDIOPULMONARY  VO2max (ml/kg/min) 25.6 28.0 91  VO2max (ml/min) 2150 2352 91  HR/VO2 (beats/L STPD) 50 50 100  HR rest (bpm) 80  HR max (bpm) 166 157 106  Max systolic BP (mmHg) 176 192 92  Vo2/Watts (ml/W) 10.0 11.0 91  Maximum R (VCo2/Vo2) 1.2 1.1 110  Anaerobic threshold (ml/min) 1050 1176 89  AT/VO2 (%) 49 50 98    RESPIRATORY  Rest ventilation (L BTPS/min) 10.0 5.0 200  Ve/VO2 mid (L BTPS/L STPD) 30.0 28.0 107  Ve/VO2 max (L BTPS/L STPD) 40.0 33.0 121  Ve/VCO2 mid (L BTPS/L STPD) 34.0 25.0 136  Ve/VCO2 max (L BTPS/L STPD) 35.0 30.0 117  Max tidal Vt (L BTPS) 2.6 2.7 96  Max ventilation (L BTPS/min) 91.0 124.0 73  Breathing Reserve (%) 27.0 30.0 90  Exercise (A-a)o2 (mmHg) 30  Exercise Vd/Vt (fraction) 0.00 0.20 0  ABG'S/SaO2 HCO3 PCO2 PO2 %SAT  Rest (Room air) 0 0 0 95  Exercise (Room air) 0 0 0 96  Max Power 170 Watts  Max RPE: 7/10  Resting blood pressure: 120/80  Exercise blood pressure: 176/90    DISCUSSION:  Pulmonary exercise testing reveals a maximum oxygen uptake of 25.6 ml/kg/min or 91% of predicted. This is within normal limits for an individual of this age and sex. The patient stopped due to dyspnea and fatigue. The Maximum RPE achieved was 7/10. The resting heart rate is within normal limits. The heart rate response to exercise is within normal limits. There is an anaerobic threshold which is indicative of a normal circulatory limitation to exercise. The maximum heart rate achieved is 166 bpm or 106% predicted. This suggests a maximum cardiovascular effort. Blood pressure is normal at rest and during exercise. The ECG is normal at rest. The exercise ECG reveals no diagnostic abnormalities. Resting spirometry  reveals airflow obstruction. No post exercise spirometry was performed. There is a normal ventilatory response to exercise. Maximum predicted ventilation is not achieved. No major abnormality of breathing pattern is noted. At rest on room air the oxygen saturation is adequate. During exercise there is no significant arterial desaturation.  CONCLUSION:  - Exercise performance is normal.  - Functionally normal exercise evaluation.  Note:  This interpretation is based on computer generated logic. It should not be used in the clinical management of the patient until confirmed by a physician.  Reviewed by:_____________________  Dr. Robert M. Ross |
| --- |
| Example Two:  Diagnosis: (Dilated Cardiomyopathy and mild Pulmonary hypertension)    Age: 49 Gender: Female Height (in) : 64.0 (cm): 162.6  BMI: 19.9 Weight (lbs): 116.0 (kg): 52.7  Exercise Time: 4:0 Barometric Pressure (mm Hg): 656  RESULTS  SPIROMETRY ACTUAL PREDICTED %PREDICTED  Pre Exercise  FEV1 (L BTPS) 2.39 2.80 85  FVC (L BTPS) 3.44 3.60 96  ratio (%) 69 78 88  MVV (L BTPS) 129 128 101    CARDIOPULMONARY  VO2max (ml/kg/min) 15.8 27.0 59  VO2max (ml/min) 832 1422 59  HR/VO2 (beats/L STPD) 200 50 400  HR rest (bpm) 85  HR max (bpm) 166 174 95  Max systolic BP (mmHg) 174 139 125  Vo2/Watts (ml/W) 6.7 11.0 61  Maximum R (VCo2/Vo2) 1.3 1.1 116  Anaerobic threshold (ml/min) 600 711 84  AT/VO2 (%) 72 50 144  RESPIRATORY  Rest ventilation (L BTPS/min) 10.0 5.0 200  Ve/VO2 mid (L BTPS/L STPD) 38.0 28.0 136  Ve/VO2 max (L BTPS/L STPD) 55.0 33.0 167  Ve/VCO2 mid (L BTPS/L STPD) 35.0 25.0 140  Ve/VCO2 max (L BTPS/L STPD) 42.0 30.0 140  Max tidal Vt (L BTPS) 1.2 2.1 58  Max ventilation (L BTPS/min) 47.0 129.0 36  Breathing Reserve (%) 64.0 30.0 213  Exercise (A-a)o2 (mmHg) 20 30 67  Exercise Vd/Vt (fraction) 0.35 0.20 175  ABG'S/SaO2 HCO3 PCO2 PO2 %SAT  Rest (Room air) 24 35 77 96  Exercise (Room air) 17 30 84 95  Max Power 80 Watts  Max RPE: 4/10  Resting blood pressure: 120/80  Exercise blood pressure: 174/87    DISCUSSION:  Pulmonary exercise testing reveals a maximum oxygen uptake of 15.8 ml/kg/min or 59% of predicted. This is markedly reduced for an individual of this age and sex. The patient stopped due to dyspnea and fatigue. The Maximum RPE achieved was 4/10. The measured VO2 max is significantly less than predicted from the external power output. This may be due to abnormal oxygen kinetics or technical measurement problems. The resting heart rate is within normal limits. The heart rate response to exercise is very high. There is evidence of an anaerobic threshold. The maximum heart rate achieved is 166 bpm or 95% predicted. This suggests a maximum cardiovascular effort. Blood pressure is normal at rest and during  exercise. The resting ECG suggests Cor Pulmonale. The exercise ECG reveals  non-specific changes. Resting spirometry reveals airflow obstruction. No post exercise spirometry was performed. There is a high ventilatory response to exercise. Maximum predicted ventilation is not achieved. There is evidence of shallow breathing. Acid base status at rest is normal. After exercise there is evidence of lactic acidosis. Blood gases at rest reveal alveolar hyperventilation with normal oxygenation. Alveolar hyperventilation increases further with exercise. There is increased "dead space" ventilation, otherwise gas exchange is normal. At rest on room air the oxygen saturation is adequate. During exercise there is no significant arterial desaturation.  CONCLUSION:  - Exercise performance is significantly reduced.  - The patient stopped due to dyspnea and fatigue.  - ECG evidence of Cor Pulmonale.  - Exercise induced minor ECG abnormalities. Appropriate further evaluation  and treatment is suggested if clinically indicated.  - Cardiopulmonary disorder leading to a low stroke volume and wasted  ventilation. Further studies to evaluate the possibility of pulmonary vascular disease or CHF are suggested if clinically indicated.  - There is a low oxygen cost relative to the external power output. This suggests abnormalities of oxygen transport mechanisms.  This patient's 8 hour work capacity is 3.5 METs. Energy requirements are approximately 2.5-3 METs for office work, 3-4 METs for domestic or light factory work, 4-6 METs for outdoor or regular factory work, and above 6 METs for heavy work. |
| Example three:  Diagnosis: (Chronic Obstructive Pulmonary Disease (COPD))    Age: 66 Gender: Male Height (in) : 69.0 (cm): 175.3  BMI: 19.8 Weight (lbs): 134.0 (kg): 60.8  Exercise Time: 8:0 Barometric Pressure (mm Hg): 656  RESULTS  SPIROMETRY ACTUAL PREDICTED %PREDICTED  Pre Exercise  FEV1 (L BTPS) 0.88 3.50 25  FVC (L BTPS) 2.44 4.50 54  ratio (%) 36 78 46  MVV (L BTPS) 38 31 123  CARDIOPULMONARY  VO2max (ml/kg/min) 17.4 26.4 66  VO2max (ml/min) 1059 1606 66  HR/VO2 (beats/L STPD) 75 50 150  HR rest (bpm) 80  HR max (bpm) 141 165 85  Max systolic BP (mmHg) 166 148 112  Vo2/Watts (ml/W) 10.0 11.0 91  Maximum R (VCo2/Vo2) 1.0 1.1 94  Anaerobic threshold (ml/min) 748 803 93  AT/VO2 (%) 71 50 141  RESPIRATORY  Rest ventilation (L BTPS/min) 17.0 5.0 340  Ve/VO2 mid (L BTPS/L STPD) 42.0 28.0 150  Ve/VO2 max (L BTPS/L STPD) 42.0 33.0 127  Ve/VCO2 mid (L BTPS/L STPD) 44.0 25.0 176  Ve/VCO2 max (L BTPS/L STPD) 42.0 30.0 140  Max tidal Vt (L BTPS) 1.3 1.5 89  Max ventilation (L BTPS/min) 46.0 38.0 121  Breathing Reserve (%) -21.0 30.0 -70  Exercise (A-a)o2 (mmHg) 28 30 93  Exercise Vd/Vt (fraction) 0.56 0.20 280  ABG'S/SaO2 HCO3 PCO2 PO2 %SAT  Rest (Room air) 24 38 65 92  Exercise (Room air) 21 46 55 83  Max Power 70 Watts  Max RPE: 10/10  Resting blood pressure: 122/75  Exercise blood pressure: 166/72    DISCUSSION:  Pulmonary exercise testing reveals a maximum oxygen uptake of 17.4 ml/kg/min or 66% of predicted. This is significantly reduced for an individual of this age and sex. The patient stopped due to dyspnea. The Maximum RPE achieved was 10/10. This suggests a maximal effort under appropriate clinical conditions. The resting heart rate is within normal limits. The heart rate response to exercise is very high. There is evidence of an anaerobic threshold. The maximum heart rate achieved is 141 bpm or 85% predicted. Blood pressure is normal at rest and during exercise. The ECG is normal at rest. The exercise ECG reveals non-specific changes. Resting spirometry reveals significant airflow obstruction. No post exercise spirometry was performed. Ventilation is elevated at rest.  There is a high ventilatory response to exercise. Maximum predicted ventilation is achieved indicating a ventilatory limitation to exercise. The respiratory frequency is elevated compatible with the reduced vital capacity. Acid base status at rest is normal. Blood gases at rest reveal normal alveolar ventilation with normal oxygenation. After exercise alveolar hypoventilation becomes manifest. There is increased "dead space" ventilation, otherwise gas exchange is normal. At rest on room air the oxygen saturation is adequate. During exercise there is marked arterial desaturation.  CONCLUSION:  - Exercise performance is significantly reduced.  - The patient stopped due to dyspnea.  - Severe obstructive airways disease with alveolar hypoventilation and hypoxemia. Supplemental oxygen during exercise may improve exercise tolerance.  - Exercise induced minor ECG abnormalities. Appropriate further evaluation  and treatment is suggested if clinically indicated.  - Reduced stroke volume compatible with unfitness or mild functional cardiac impairment. Aerobic training may improve exercise tolerance if there is no evidence of significant valvular or primary myocardial disease.  - There is a ventilatory limitation to exercise. This is abnormal.  This patient's 8 hour work capacity is 2.5 METs. Energy requirements are approximately 2.5-3 METs for office work, 3-4 METs for domestic or light factory work, 4-6 METs for outdoor or regular factory work, and above 6 METs for heavy work. |
| Example Four:  Diagnosis: (Interstitial Pulmonary Fibrosis)    Age: 72 Gender: Male Height (in) : 67.0 (cm): 170.2  BMI: 27.6 Weight (lbs): 176.0 (kg): 79.9  Exercise Time: 6:0 Barometric Pressure (mm Hg): 722  RESULTS  SPIROMETRY ACTUAL PREDICTED %PREDICTED  Pre Exercise  FEV1 (L BTPS) 1.70 2.60 65  FVC (L BTPS) 2.34 3.90 60  ratio (%) 73 67 109  MVV (L BTPS) 61 91 67  CARDIOPULMONARY  VO2max (ml/kg/min) 14.9 24.0 62  VO2max (ml/min) 1191 1918 62  HR/VO2 (beats/L STPD) 61 50 122  HR rest (bpm) 70  HR max (bpm) 132 163 81  Max systolic BP (mmHg) 172 154 112  Vo2/Watts (ml/W) 10.0 11.0 91  Maximum R (VCo2/Vo2) 1.0 1.1 95  Anaerobic threshold (ml/min) 799 959 83  AT/VO2 (%) 67 50 134  RESPIRATORY  Rest ventilation (L BTPS/min) 15.0 5.0 300  Ve/VO2 mid (L BTPS/L STPD) 40.0 28.0 143  Ve/VO2 max (L BTPS/L STPD) 46.0 33.0 139  Ve/VCO2 mid (L BTPS/L STPD) 49.0 25.0 196  Ve/VCO2 max (L BTPS/L STPD) 48.0 30.0 160  Max tidal Vt (L BTPS) 1.3 1.4 93  Max ventilation (L BTPS/min) 57.0 61.0 93  Breathing Reserve (%) 7.0 30.0 23  Exercise (A-a)o2 (mmHg) 52 30 173  Exercise Vd/Vt (fraction) 0.55 0.20 275  ABG'S/SaO2 HCO3 PCO2 PO2 %SAT  Rest (Room air) 25 42 72 94  Exercise (Room air) 21 42 49 80  Max Power 95 Watts  Max RPE: 6/10  Resting blood pressure: 120/80  Exercise blood pressure: 172/86    DISCUSSION:  Pulmonary exercise testing reveals a maximum oxygen uptake of 14.9 ml/kg/min or 62% of predicted. This is significantly reduced for an individual of this age and sex. The patient stopped due to dyspnea and leg fatigue. The Maximum RPE achieved was 6/10. The resting heart rate is within normal limits. The heart rate response to exercise is high. There is evidence of an anaerobic threshold. The maximum heart rate achieved is 132 bpm or 81% predicted. Blood pressure is normal at rest and during exercise. The ECG is normal at rest. The exercise ECG reveals no diagnostic abnormalities. Resting spirometry reveals restriction. No post exercise spirometry was performed. Ventilation is elevated at rest. There is a high ventilatory response to exercise. Ventilatory reserve is significantly diminished. The respiratory frequency is elevated compatible with the reduced vital capacity. Acid base status at rest is normal. After  exercise there is evidence of lactic acidosis. Blood gases at rest reveal normal alveolar ventilation with normal oxygenation. There is no significant abnormality of alveolar ventilation with exercise. Gas exchange becomes abnormal with increased areas of both high and low V/Q ("dead space" and "shunt" fraction) leading to wasted ventilation and pulmonary circulation. At rest on room air the oxygen saturation is adequate. During exercise there is marked arterial desaturation.  CONCLUSION:  - Exercise performance is significantly reduced.  - The patient stopped due to dyspnea and leg fatigue.  - Cardiopulmonary disorder leading to a low stroke volume and gas exchange  abnormalities. Further studies to evaluate the possibility of interstitial/alveolar lung disease and/or pulmonary vascular disease are suggested if indicated.  This patient's 8 hour work capacity is 2.0 METs. Energy requirements are approximately 2.5-3 METs for office work, 3-4 METs for domestic or light factory work, 4-6 METs for outdoor or regular factory work, and above 6 METs for heavy work. |
| Example Five:  Diagnosis: (Pulmonary vascular disease)    Age: 22 Gender: Male Height (in) : 73.0 (cm): 185.4  BMI: 22.4 Weight (lbs): 170.0 (kg): 77.2  Exercise Time: 11:0 Barometric Pressure (mm Hg): 656  RESULTS  SPIROMETRY ACTUAL PREDICTED %PREDICTED  Pre Exercise  FEV1 (L BTPS) 3.41 4.20 81  FVC (L BTPS) 3.65 4.50 81  ratio (%) 93 93 100  MVV (L BTPS) 156 168 93  CARDIOPULMONARY  VO2max (ml/kg/min) 25.6 42.0 61  VO2max (ml/min) 1976 3242 61  HR/VO2 (beats/L STPD) 58 50 116  HR rest (bpm) 80  HR max (bpm) 176 195 90  Max systolic BP (mmHg) 174 185 94  Vo2/Watts (ml/W) 9.2 11.0 84  Maximum R (VCo2/Vo2) 1.3 1.1 115  Anaerobic threshold (ml/min) 1050 1621 65  AT/VO2 (%) 53 50 106  RESPIRATORY  Rest ventilation (L BTPS/min) 10.0 5.0 200  Ve/VO2 mid (L BTPS/L STPD) 42.0 28.0 150  Ve/VO2 max (L BTPS/L STPD) 68.0 33.0 206  Ve/VCO2 mid (L BTPS/L STPD) 42.0 25.0 168  Ve/VCO2 max (L BTPS/L STPD) 50.0 30.0 167  Max tidal Vt (L BTPS) 1.8 2.2 82  Max ventilation (L BTPS/min) 125.0 156.0 80  Breathing Reserve (%) 20.0 30.0 67  Exercise (A-a)o2 (mmHg) 47 30 157  Exercise Vd/Vt (fraction) 0.41 0.20 205  ABG'S/SaO2 HCO3 PCO2 PO2 %SAT  Rest (Room air) 22 36 67 91  Exercise (Room air) 17 29 58 87  Max Power 150 Watts  Max RPE: 10/10  Resting blood pressure: 130/91  Exercise blood pressure: 174/86      DISCUSSION:  Pulmonary exercise testing reveals a maximum oxygen uptake of 25.6 ml/kg/min or 61% of predicted. This is moderately reduced compatible with a mild functional aerobic impairment for an individual of this age and sex. The patient stopped due to dyspnea. The Maximum RPE achieved was 10/10. This suggests a maximal effort under appropriate clinical conditions. The resting heart rate is within normal limits. The heart rate response to exercise is within normal limits. There is an anaerobic threshold which is indicative of a significant cardiovascular limitation. The maximum heart rate achieved is 176 bpm or 90% predicted. This suggests a maximum cardiovascular effort. Blood pressure is normal at rest and during exercise. The resting ECG suggests Cor Pulmonale. Resting spirometry  does not reveal any functionally significant abnormalities. No post exercise spirometry was performed. There is a high ventilatory response to exercise. Maximum predicted ventilation is not achieved. No major abnormality of breathing pattern is noted. Acid base status at rest is normal. After exercise there is evidence of lactic acidosis. Blood gases at rest reveal normal alveolar ventilation with normal oxygenation. Alveolar hyperventilation occurs with exercise. Gas exchange becomes abnormal with increased areas of both high and low V/Q ("dead space" and "shunt" fraction) leading to wasted ventilation and pulmonary circulation. At rest on room air the oxygen saturation is adequate. During exercise there is significant arterial desaturation.  CONCLUSION:  - Exercise performance is significantly reduced.  - The patient stopped due to dyspnea.  - ECG evidence of Cor Pulmonale.  - Cardiopulmonary disorder leading to a low stroke volume and gas exchange  abnormalities. Further studies to evaluate the possibility of interstitial/alveolar lung disease and/or pulmonary vascular disease are suggested if indicated.  This patient's 8 hour work capacity is 4.0 METs. Energy requirements are approximately 2.5-3 METs for office work, 3-4 METs for domestic or light factory work, 4-6 METs for outdoor or regular factory work, and above 6 METs for heavy work. |

Output from the interpretive program XINT of the five patient examples discussed in the ATS/ACCP document on Cardiopulmonary Exercise Testing [1]. Please see these examples which are listed in that document for the CPET raw data and graphs.
